# *Toxocara canis* and the allergic process

**DOI:** 10.1590/0074-02760150051

**Published:** 2015-09

**Authors:** Mauricio Grecco Zaia, Sandra Regina Pereira de Oliveira, Cynthia Aparecida de Castro, Edson Garcia Soares, Ana Afonso, Luis Gustavo S Monnazzi, Oscar Peitl, Lúcia Helena Faccioli, Fernanda de Freitas Anibal

**Affiliations:** 1 Universidade Federal de São Carlos, Centro de Ciências Biológicas e da Saúde, Departamento de Morfologia e Patologia, Laboratório de Parasitologia, São Carlos, SP, Brasil; 2 Universidade Federal de São Carlos, Centro de Ciências Biológicas e da Saúde, Departamento de Ciências Fisiológicas, São Carlos, SP, Brasil; 3 Universidade de São Paulo, Faculdade de Medicina de Ribeirão Preto, Departamento de Patologia e Medicina Legal, Ribeirão Preto, SP, Brasil; 4 Universidade Nova de Lisboa, Instituto de Higiene e Medicina Tropical, Unidade de Parasitologia Médica, Lisboa Portugal; 5 Universidade de São Paulo, Instituto de Química de São Carlos, Grupo de Bioanalítica, Microfabricação e Separações, São Carlos, SP, Brasil; 6 Centro Universitário de Araraquara, Centro de Ciências Biológicas e da Saúde, Araraquara, SP, Brasil; 7 Universidade Federal de São Carlos, Curso de Engenharia de Materiais, Departamento de Engenharia de Materiais, São Carlos, SP, Brasil; 8 Universidade de São Paulo, Faculdade de Ciências Farmacêuticas de Ribeirão Preto, Departamento de Análises Clínicas, Toxicológicas e Bromatológicas, Ribeirão Preto, SP, Brasil

**Keywords:** toxocariasis, ELISA, eosinophils, IgE, lungs

## Abstract

The protective effect of infectious agents against allergic reactions has been thoroughly investigated. Current studies have demonstrated the ability of some helminths to modulate the immune response of infected hosts. The objective of the present study was to investigate the relationship between *Toxocara canis* infection and the development of an allergic response in mice immunised with ovalbumin (OVA). We determined the total and differential blood and bronchoalveolar lavage fluid cells using BALB/c mice as a model. To this end, the levels of interleukin (IL)-4, IL-5 and IL-10 and anti-OVA-IgE were measured using an ELISA. The inflammatory process in the lungs was observed using histology slides stained with haematoxylin and eosin. The results showed an increase in the total number of leukocytes and eosinophils in the blood of infected and immunised animals at 18 days after infection. We observed a slight lymphocytic inflammatory infiltrate in the portal space in all infected mice. Anti-OVA-IgE levels were detected in smaller proportions in the plasma of immunised and infected mice compared with mice that were only infected. Therefore, we concluded that *T. canis* potentiates inflammation in the lungs in response to OVA, although anti-OVA-IgE levels suggest a potential reduction of the inflammatory process through this mechanism.

*Toxocara canis* is an intestinal nematode that affects dogs. In humans, this geohelminth induces visceral larva migrans (VLM) syndrome, which is associated with severe eosinophilia, increased serum IgE and inflammation of the airways (Rogerio et al. 2003). Humans become infected after ingestion of the embryonated eggs, primarily in public parks and sandboxes that have been contaminated with animal faeces. The larvae are released into the intestinal walls and migrate to different organs, including the liver and lungs (Pinelli et al. 2007), causing fever, hepatosplenomegaly and respiratory dysfunction such as cough, wheezing and air flow obstruction (Qualizza et al. 2009). *T. canis*larvae induce a T-helper (Th)2 response, resulting in the secretion of interleukin (IL)-4 and the subsequent production of IgE and IL-5, as well as the differentiation and activation of eosinophils (Qualizza et al. 2009). Helminthic infections and allergic reactions have long been described as similar. The increase in the prevalence of allergic diseases/reactions has been associated with a reduction of infections primarily in developed countries, according to the hygiene hypothesis (Moncayo & Cooper 2006). The environment shows great influence in the hyper reactivity of the lungs and models the reactions in this organ (Wilson et al. 2005). There is robust evidence demonstrating immune suppression during helminthic infections (Medeiros Jr et al. 2003, Erb 2009). Epidemiological data have demonstrated that *Schistosoma* spp infections have great protective effects against allergic affections and *Necator americanus* protects against asthma (Okada et al. 2010). Thus, parasitic infections enhance IL-10 production, which, in turn, is inversely associated with allergic sensibility. A high IgE concentration serves as an indicator of the development of allergic disease in neonates and is an indicator of prognosis in adults with certain chronic allergic diseases (Sorensen & Sakali 2006). *T. canis* has also been studied as a potential allergy suppressor. Although some studies have indicated that *T. canis* infection exacerbates allergic reactions (Buijs et al. 1997, Wohlleben et al. 2004, Pinelli et al. 2007), additional studies are needed to better understand and confirm these findings. In the present study, we established the relationship between *T. canis* infection and lung hyperreactivity in BALB/c mice immunised with ovalbumin (OVA). The number of leukocytes (polymorphonuclear and mononuclear cells and eosinophils) in the blood and bronchoalveolar lavage fluid (BALF) was counted and serum IL-4, IL-5, IL-10 and OVA-IgE levels were determined. Pulmonary inflammation was evaluated using histological sections stained with haematoxylin and eosin (H&E). These new data will be of great importance to corroborate the relationship between*T. canis* and allergy and confirm previous results obtained in animal models for allergy using OVA.

## MATERIALS AND METHODS

*Animals* - Female BALB/c specific pathogens free mice at six-eight weeks of age and weighing 15-20 g were obtained from the animal facilities of the School of Pharmaceutical Sciences of Ribeirão Preto, University of São Paulo, Brazil. These animals were maintained under standard laboratory conditions throughout the experimental period at the Laboratory of Parasitology, Department of Morphology and Pathology, Federal University of São Carlos (UFSCar), Brazil, with free access to water and food. This project was approved by the Ethical Committee on Animal Use of UFSCar (CEA 056/2011).

*Mice infection with T. canis* - *T. canis* eggs were obtained according to the method of Olson and Schutz (1963) with modifications, according to Faccioli et al. (1996). Briefly, pregnant female worms were recovered from infected dogs and the eggs were collected from the uterus of these worms. Subsequently, the eggs were washed and incubated at 37ºC in 2% formalin to facilitate progression to the infectious stage. On day 0, 12 mice were infected through an intragastric route with 0.2 mL of saline containing 500 embryonated*T. canis* eggs.

*Immunisation and challenge with OVA* - Animal immunisation was performed on days 0 and 7 through the subcutaneous injection of 4 μg of OVA and 1.6 mg aluminium hydroxide in 0.4 mL saline. All animals were challenged twice through an intranasal route (at 12 and 17 days post-immunisation) with 10 μg of OVA in 50 μL of saline, delivered into the nostrils. All assays were performed at 24 h after the second challenge [at 18 days post-infection (p.i.)] and six mice from each group were sacrificed. Two sets of experiments were performed under the same conditions (Russo et al. 2001). There were four groups per experiment: control (challenged with OVA at 12 and 17 days p.i.), OVA (immunised subcutaneously on days 0 and 7 and challenged with OVA at 12 and 17 days p.i.), *T. canis* (infected with *T. canis* on day 0 and challenged with OVA at 12 and 17 days p.i.) and OVA + *T. canis*(infected with *T. canis* on day 0, immunised subcutaneously on days 0 and 7 and challenged with OVA at 12 and 17 days p.i.) (Table).

**TABLE:** Experimental design for immunisation and challenge with ovalbumin (OVA)

Experimental group	*Toxocara canis * infection	OVA immunisation	OVA challenge
Control	No	No	12th and 17th p.i.
OVA	No	0 and 7th p.i.	12th and 17th p.i.
*T. canis*	Yes	No	12th and 17th p.i.
OVA + *T. canis*	Yes	0 and 7th p.i.	12th and 17th p.i.

p.i.: post-infection.

*Extraction of fluids and cell counts* - The mice were anaesthetised with sodium pentobarbitone (30 mg/kg intravenous) and the peripheral blood (PB) samples were obtained through cardiac puncture. Absolute leukocyte counts were measured after counting in a Neubauer chamber. PB was collected using ethylenediamine tetraacetic acid (EDTA) as an anticoagulant. The absolute number of different leukocytes (mononuclear and polymorphonuclear cells and eosinophils) was obtained from differential counts on blood smears stained with Panótico Rápido LB (Laborclin Ltda, Brazil).

The cells in the peritoneal cavity (PC) were collected after injection with 3 mL of phosphate-buffered saline (PBS) containing 0.5% sodium citrate. To collect the BALF, a polyethylene cannula was introduced in the trachea and 1 mL of PBS/sodium citrate was injected. These procedures were repeated twice to obtain a greater number of cells. The total number of leukocytes in the PC and BALF was counted in a Neubauer chamber and the differential count was obtained from slides prepared using cytospin (SEROCITO mod. 2400; FANEM, Brazil) (1,000 rpm/3 min) and stained with Panótico Rápido LB.

*Cytokines* - Commercially available antibodies for the ELISA were used to measure the levels of IL-4, IL-5 and IL-10 in the plasma according to the manufacturer’s instructions (BD OptEIA™; BD Biosciences).

*IgE anti-OVA* - ELISAs were performed according to the methods ofSchnare et al. (2001) and (Rogerio et al. 2003) with modifications. Polystyrene microtitration plates (Greiner Bio-One) were coated with OVA, Chicken E19 Soma A-5253 (100 μL/well), at a concentration of 10 μg/mL in 0.1 M carbonate-bicarbonate buffer (CBB), pH 9.6, for 18 h at 4ºC. The plates were blocked with 10% skimmed milk in 1X PBS for 2 h at room temperature (RT) (200 μL/well) and washed three times with PBS-T (PBS + 0.1% Tween 20) using a microplate washer (Bras Serum, model BS II). The serum was diluted (1:1) in PBS and incubated for 2 h at room temperature (100 μL/well), followed by washing three times. Anti-mouse IgE monoclonal antibody (BD Biosciences) in CBB was added (100 μL/well) and incubated for 1 h. After washing, biotinylated anti-mouse IgE monoclonal antibody in 1X PBS was added (100 μL/well) and incubated for 1 h. Streptavidin-horseradish peroxidase conjugate was added and incubated for 1 h (100 μL/well). Subsequently, the reaction was developed for 20 min using a 3,3′,5,5′-tetramethylbenzidine substrate (50 μL/well) and terminated after the addition of 50 μL 0.1 M sulphuric acid to each well. The absorbance at 450 nm was measured using an automatic microplate reader (Tecan SLT Spectra).

*Histology* - The lungs were removed from the mice and immediately fixed in 10% formalin at 18 days after infection. The specimens were routinely processed, embedded in paraffin blocks and sectioned into 5 μm thick sections followed by staining with H&E for examination under light microscopy. The slides were photographed at 100X and 500X magnification using a Leica DMRX microscope equipped with a suitable camera.

*Statistical analysis* - Each experiment was performed twice and the data analysis was performed using one-way ANOVA, followed by Bonferroni’s correction for multiple comparisons, using GraphPad Prism 5. Differences were considered significant at a p value < 0.05.

## RESULTS

**Fig. 1:**
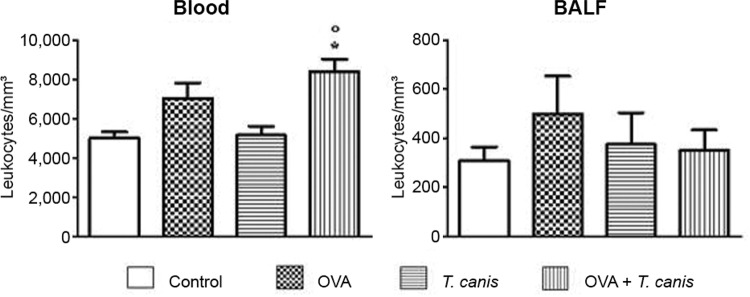
: total number of leukocytes in blood and bronchoalveolar lavage fluid (BALF). On the 18th day after* Toxocara canis*infection, the animals were sacrificed and the different biological materials were obtained and analysed. The data represent the mean ± standard deviation (n = 6 animals) of two independent experiments. Significant compared to control (*) and *T. canis* (°). Differences were considered significant when p < 0.05. OVA: ovalbumin.

*Leukocytes* - The number of leukocytes in the blood of the OVA +*T. canis* group was significantly higher than that in the other experimental groups, except OVA (p < 0.05). In the BALF, no significant difference was observed (Fig. 1).

*Mononuclear and polymorphonuclear cells* - Analysis of the mononuclear cells present in the blood showed that OVA + *T. canis*was the only experimental group that displayed a significant increase in cell number compared with the other three experimental groups (p < 0.05). In the BALF,*T. canis* and OVA + *T. canis* experimental groups presented a significant increase in mononuclear cells compared with the OVA group (p < 0*.*05). No significant relationship between the other groups was observed.

**Fig. 2:**
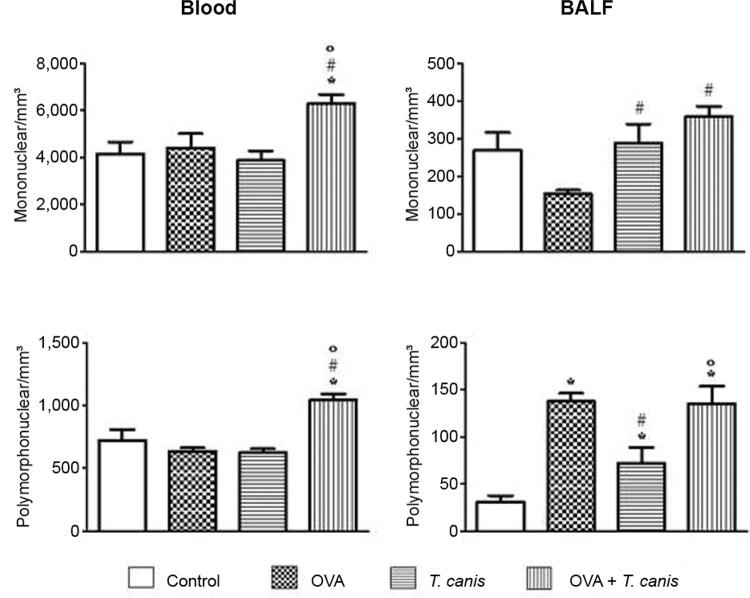
: total number of mononuclear and polymorphonuclear in blood and bronchoalveolar lavage fluid (BALF). On the 18th day after*Toxocara canis* infection the animals were sacrificed and the different biological materials were obtained and analysed. The data represent the mean ± standard deviation (n = 6 animals) of two independent experiments. Significant compared to control (*), ovalbumin (OVA) (#) and *T. canis* (°). Differences were considered significant when p < 0.05.

There was no statistically significant difference in the number of blood polymorphonuclear cells between the control, OVA and *T. canis*experimental groups (p > 0.05). However, the OVA + *T. canis* group showed an increase in the number of polymorphonuclear cells compared with the other experimental groups. In the BALF, the number of polymorphonuclear cells in all experimental groups was significantly higher compared with the control group (p < 0.05). Although the values for *T. canis* were higher than in the control group, these values were significantly lower compared with the OVA and OVA + *T. canis*experimental groups (Fig. 2).

**Fig. 3:**
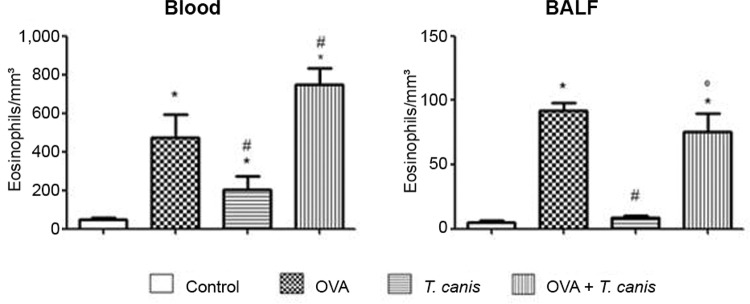
: total number of eosinophils in blood and bronchoalveolar lavage fluid (BALF). On the 18th day after *Toxocara canis*infection the animals were sacrificed and the different biological materials were obtained and analysed. The data represent the ± standard deviation (n = 6 animals) of two independent experiments. Significant compared to control (*), ovalbumin (OVA) (#) and *T. canis* (°). Differences were considered significant when p < 0.05.

*Eosinophils* - In the blood, we observed an increase in the eosinophil number in all groups compared with the control group. Eosinophils cell numbers obtained in *T. canis* experimental group was lower than in the OVA group. Animals also presented an increase in eosinophils in the OVA +*T. canis* group compared with the OVA and *T. canis* groups. The results obtained from the BALF revealed that OVA and OVA + *T. canis* groups presented a significant increase in eosinophil numbers compared with the values obtained in the control and *T. canis* groups (p < 0.05). Although not significant, a slight reduction in the eosinophil number was observed in OVA + *T. canis*group compared with the OVA group in the BALF (p* > *0.05). These results clearly suggest that the primary inducer of eosinophilia in this model was OVA, as the OVA group presented a higher number of these cells compared with the*T. canis* and OVA + *T. canis* groups (Fig. 3).

*Cells from the PC* - The mononuclear and polymorphonuclear cells and PC eosinophils were obtained and counted. No significant differences were observed among the experimental groups, regardless of cell type.

**Fig. 4:**
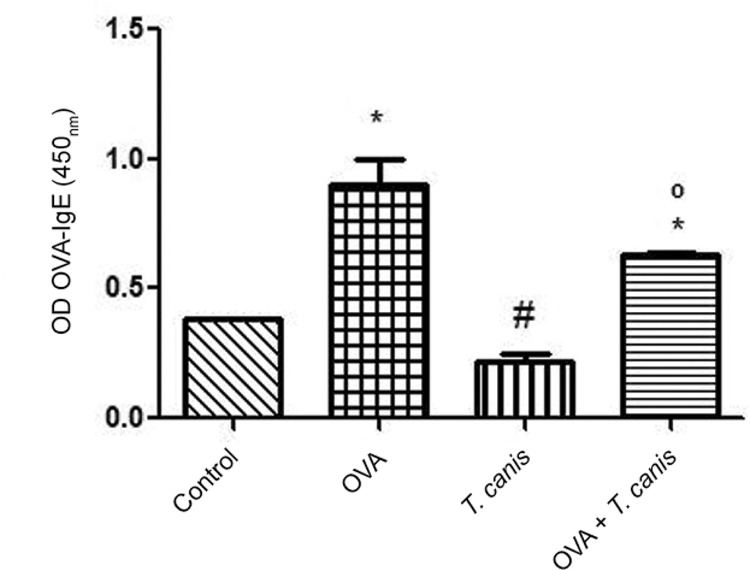
: ovalbumin (OVA)-IgE measure. Polystyrene microtitration plates (Greiner Bio-One) were coated with OVA, Chicken E19 Soma A-5253 (100 µL/well), at a concentration of 10 µg/mL and an ELISA was realised to measure OVA-IgE in plasma of BALB/c mice. The results are shown as optical density (OD) OVA-IgE. Data represent triplicates of pooled plasma obtained 18 days after infection. Significant compared to control (*), OVA (#) and *Toxocara canis* (°). Differences were considered significant when p < 0.05.

*OVA-IgE - *The data obtained after measuring the OVA-IgE concentration showed that the animals infected with *T. canis*presented a decrease in the levels of OVA-IgE compared with the uninfected groups. The OVA + *T. canis* group showed a 1.4-fold decrease in the levels of OVA-IgE compared with the OVA group. In addition, the *T. canis*group also showed a 1.7-fold decrease compared with the control group (Fig. 4).

*IL levels* - The levels of IL-4, IL-5 and IL-10 were measured in mice plasma from all experimental groups using an ELISA. No changes were observed among the levels of measured ILs from all groups (data not shown).

**Fig. 5:**
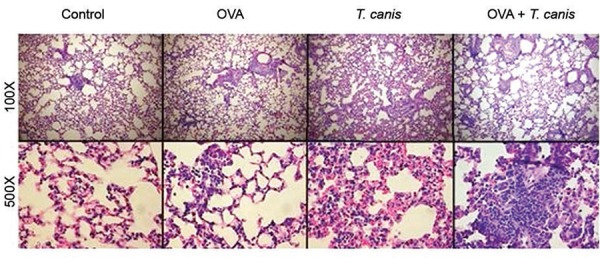
: lung histology stained with haematoxylin and eosin. Control, ovalbumin (OVA), *Toxocara canis *and OVA + *T. canis* groups are presented with 100X and 500X magnification.

*Histology* - To determine whether the inhibition of the inflammatory response and down-modulation of pathology resulted from infection, lung histological sections were compared between the OVA and OVA + *T. canis*experimental groups. The lung histological sections from the OVA experimental group presented peribronchial inflammation, predominantly lymphocyte in nature, with vascular congestion. Neutrophils and some eosinophils remained present throughout the lung parenchyma. Animals from OVA + *T. canis* experimental group displayed a more severe inflammatory peribronchial and intraseptal lymphocytic process. The predominance of lymphocytes and neutrophils and the widening of the alveolar septa were observed. The control group presented preserved cellular structures with rare inflammatory sites, presenting neutrophils and lymphocytes and occasional foci of intraalveolar haemorrhage. The *T. canis* group presented a much larger vascular congestion compared with the control and OVA experimental groups. Dense septal lengthening inflammatory infiltrate predominantly comprising lymphocytes, plasma cells and a few neutrophils were observed (Fig. 5).

## DISCUSSION

Previous studies have demonstrated that helminthic infections might modulate a Th2 response in allergic patients (Sorensen & Sakali 2006, Ponte et al. 2007, Okada 2010). In the present study, OVA was used as an allergy model to determine whether*T. canis* infection modulates and decreases the allergic inflammation response stimulated through OVA in BALB/c mice. Analyses of the cells in the blood and BALF showed interesting results. A comparison of the data obtained from the OVA and OVA + *T. canis* experimental groups showed an increase in all cell types in the infected group. The increase in the eosinophil numbers in the *T. canis* group might reflect the increased stimulation induced through secretion/excretion antigens from *T. canis* larvae. *Toxocara* sp. larvae secrete antigens with allergenic properties, suggesting that this parasite may generate high levels of total IgE (Roldán 2010). Some*Toxocara* sp. antigens induce a Th2 response with IL-4 production and subsequent differentiation of B-lymphocytes into plasma cells secreting IgE antibodies and IL-5 for the differentiation and activation of eosinophils (Qualizza et al. 2009). We hypothesised that the eosinophils present in the BALF primarily reflect OVA, because mice from the *T. canis *experimental group did not present this cell type. This result suggests that infection with *T. canis* stimulates the influx of eosinophils into PB in BALB/C mice, but exerts no influence in the BALF. Mononuclear cells were predominantly observed in the blood from mice of the OVA + *T. canis* experimental group, indicating that when synergistically stimulated (OVA and *T. canis* infection), the function of lymphocytes is primarily induced, reflecting an increase in reactivity and the release of antigens. In addition, this group also showed the highest influx of leukocytes in BALF. We observed a significant increase on polymorphonuclear cell in the blood of OVA + *T. canis *experimental group compared with other experimental groups. In the BALF, this increase was observed in all groups compared with the control. OVA and OVA + *T. canis* experimental groups presented a higher number of polymorphonuclear compared with *T. canis* experimental group, suggesting that both *T. canis* larvae and particularly OVA stimulate the influx of polymorphonuclear cells into this site.

No differences in the levels of IL-4, IL-5 and IL-10 were observed (data not shown). Thus, we concluded that infection with both OVA and *T. canis*similarly stimulated the production of these cytokines and when animals were treated with both stimuli (OVA and *T. canis *infection), the response pattern did not significantly change in this experimental design. Pinelli et al. (2005) showed that IL-4 and IL-10 levels in the BALF did not change during *T. canis*experimental infection, consistent with the results of the present study. Perhaps these results would have been different if the cytokine levels were evaluated through reverse transcription-polymerase chain reaction (RT-PCR). Post-transcriptional modifications might occur on mRNA, thereby compromising protein synthesis. ELISA results are not always consistent with RT-PCR data, although ELISA results are more conclusive. An interesting finding in the present study was obtained from the analysis of OVA-IgE. We observed a 1.4-fold decrease in OVA-IgE levels in OVA + *T. canis* mice compared with the OVA group. In the*T. canis* group, a 1.7-fold decrease was observed compared with the control group. This result strongly suggested that *T. canis*infection induced a decrease in OVA-IgE production and might reduce the release of anti-specific allergen (OVA). However, it was not possible to identify a decrease in inflammation in general, likely reflecting the increase in total IgE and the anti-soluble IgE antigens of *T. canis*. The results of the present study demonstrated that *T. canis* infection leads to damage in the lung epithelium, with peribronchial inflammation, eosinophil influx and widening of the alveolar septa. Most of the histological findings obtained from the lungs were consistent with previous data showing alterations in lung tissue associated with*T. canis* infection (Pinelli et al. 2006). We proposed that much of the damage reported in the present study is associated with *T. canis* infection, indicating that infection exacerbates the symptoms and the damage of the lung tissue, even under these experimental conditions. Previous studies with *Heligmosomoides polygyrus* have shown that parasites might suppress inflammation in allergic airways induced through OVA. Moreover, in models of chronic infection with*Schistosoma mansoni,* allergic reactions induced through OVA promote a decrease in pulmonary eosinophilia (van Riet et al. 2007). Inconsistencies among studies with helminths might reflect several factors: age, genetics and helminth species (van Riet et al. 2007). Other studies have demonstrated that the type of infection might interfere with the response pattern. The results of a population study in Ethiopia showed that an intense infection with*Ascaris* sp. might contribute to the decreased wheezing in children (Dagoye et al. 2003). In addition, chronic helminthic infection is an important factor in the suppression of allergic inflammation (Moncayo & Cooper 2006). However, the infection with *T. canis *was not associated with an improvement in chronic OVA-induced allergic manifestation (Pinelli et al. 2005). The results of the present study strongly suggested that acute infection is also unable to improve inflammation, even using a high parasite load and with a decrease in OVA-IgE. Differences in the results reported in previous studies conducted with *T. canis* might reflect the use of variant parasite specimens obtained in various parts of the world. Differences and similarities have been observed in *Toxocara* sp. (Chen et al. 2012), suggesting that further investigation of the strains used in the present study is needed. Thus, the characterisation of the strains/lines of *Toxocara* might be useful for understanding this disease and in studies using the VLM model.

Several studies have suggested that some helminthic infections show therapeutic potential against immunopathology diseases. The results of the present study demonstrated that *T. canis* infection exacerbated experimental airway allergic inflammation in an acute infection model (18 days), which is consistent with previous data from human epidemiological studies and other animal models.
